# *Graph* *T*–*T* (V1.0Beta), a program for embedding and visualizing periodic graphs in 3D Euclidean space

**DOI:** 10.1107/S2053273324002523

**Published:** 2024-04-29

**Authors:** Maxwell Christopher Day, Ali Rostami, Frank Christopher Hawthorne

**Affiliations:** a Department of Earth Sciences, University of Manitoba, Winnipeg, Manitoba R3T 2N2, Canada; b Computer Science Department, Friedrich Schiller University Jena, Jena, 07743, Germany; Universidad del País Vasco, Spain

**Keywords:** bond topology, chains of tetrahedra, (SiO_4_)^4−^ tetrahedra, graph embedding program, 3D Euclidean space, 3D spring-force algorithm, *Graph* *T*–*T*

## Abstract

Following the work of Day & Hawthorne [*Acta Cryst.* (2022), A**78**, 212–233] and Day *et al.* [*Acta Cryst.* (2024), A**80**, 258–281], the program *Graph* *T*–*T* has been developed to embed graphical representations of observed and hypothetical chains of (SiO_4_)^4−^ tetrahedra into 2D and 3D Euclidean space. During embedding, the distance between linked vertices (*T*–*T* distances) and the distance between unlinked vertices (*T*⋯*T* separations) in the resultant unit-distance graph are restrained to the average observed distance between linked Si tetrahedra (3.06±0.15 Å) and the minimum separation between unlinked vertices is restrained to be equal to or greater than the minimum distance between unlinked Si tetrahedra (3.713 Å) in silicate minerals. The notional interactions between vertices are described by a 3D spring-force algorithm in which the attractive forces between linked vertices behave according to Hooke’s law and the repulsive forces between unlinked vertices behave according to Coulomb’s law. Embedding parameters (*i.e.* spring coefficient, *k*, and Coulomb’s constant, *K*) are iteratively refined during embedding to determine if it is possible to embed a given graph to produce a unit-distance graph with *T*–*T* distances and *T*⋯*T* separations that are compatible with the observed *T*–*T* distances and *T*⋯*T* separations in crystal structures. The resultant unit-distance graphs are denoted as *compatible* and may form crystal structures if and only if all distances between linked vertices (*T*–*T* distances) agree with the average observed distance between linked Si tetrahedra (3.06±0.15 Å) and the minimum separation between unlinked vertices is equal to or greater than the minimum distance between unlinked Si tetrahedra (3.713 Å) in silicate minerals. If the unit-distance graph does not satisfy these conditions, it is considered *incompatible* and the corresponding chain of tetrahedra is unlikely to form crystal structures. Using *Graph* *T*–*T*, Day *et al.* [*Acta Cryst.* (2024), A**80**, 258–281] have shown that several topological properties of chain graphs influence the flexibility (and rigidity) of the corresponding chains of Si tetrahedra and may explain why particular compatible chain arrangements (and the minerals in which they occur) are more common than others and/or why incompatible chain arrangements do not occur in crystals despite being topologically possible.

## Abstract 2

The program *Graph* *T*–*T* (V1.0Beta) has been developed to embed graphical representations of observed and hypothetical chains of (SiO_4_)^4−^ tetrahedra into 2D and 3D Euclidean space.

## 1. Introduction

*Graph* *T*–*T* (V1.0Beta) is a user-friendly program for embedding finite and/or periodic graphs in 3D Euclidean space to produce unit-distance graphs while restraining several metric properties. These metric properties (*e.g.* edge lengths) are calculated in real time during the embedding process to allow a better understanding of how the topological properties of the input graph affect the geometrical properties of the corresponding unit-distance graph. Day *et al.* (2024 ▸) used *Graph* *T*–*T* extensively to understand what topological properties of 1-periodic arrangements of (*T*O_4_) tetrahedra control the compatibility of such arrangements with the metrics of chain arrangements observed in chain-silicate minerals and related synthetic compounds.

There has been much work developing software and programming languages for generating and manipulating graphs and for calculating their graph-theoretic properties: e.g. Wolfram Language (graphs and matrices); *MATLAB* (Menke & Menke, 2022 ▸); *Sage* (Joyner, 2007 ▸); Java (*JGraphT*) (Michail *et al.*, 2019 ▸); C++ (*Boost Graph Library*) (Siek *et al.*, 2002 ▸); Python (*NetworkX*) (Hagberg *et al.*, 2008 ▸). However, such software and programming languages have limited options with regards to embedding graphs while simultaneously restraining their metric properties. Instead, they focus on manipulation of graphs via their corresponding adjacency matrices and calculation of various properties of the graphs.

Software programs such as *Systre* (Delgado-Friedrichs & O’Keeffe, 2003 ▸) and *ToposPro* (Blatov *et al.*, 2014 ▸) have been designed specifically for the topological and geometrical analysis of periodic nets observed in crystal structures. These programs are linked to databases of 3-, 2- and 1-periodic nets [*e.g.* the Reticular Chemistry Structure Resource (RCSR) and the Topological Types Database (TTD)]. However, such databases contain a limited number of 1-periodic graphs, for example the RCSR database contains only 11 1-periodic graphs compared with the ∼1500 non-isomorphic 1-periodic graphs generated by Day & Hawthorne (2022 ▸). The *Systre* program can also be used to embed periodic nets represented as labelled *quotient graphs*. Methods related to the use of quotient graphs (*e.g.* Treacy *et al.*, 1997 ▸, 2004 ▸) have been used to generate 0- to 3-periodic structures related to zeolitic silicates including 1-periodic rods and tubes (Treacy *et al.*, 2023 ▸). Other types of 1-periodic structures, unrelated to silicate structures, have also been generated and described using analogous methods (O’Keeffe & Treacy, 2021 ▸, 2022 ▸).

However, testing of the quotient-graph methods for describing and generating periodic nets (*e.g.* Chung *et al.*, 1984 ▸; Eon, 1998 ▸, 1999 ▸; Klee, 2004 ▸) compared with the methods used by Day & Hawthorne (2022 ▸) revealed several problems. For some vertex connectivities (*i.e. ^c^V_r_*), quotient-graph methods do not generate all possible non-isomorphic nets. For example, for vertex connectivity ^2^ *V*_1_^3^ *V*_2_, the quotient-graph method for generating periodic nets [described by Chung *et al.* (1984 ▸)] produced three non-isomorphic 1-periodic nets (chain graphs) compared with the six non-isomorphic ^2^ *V*_1_^3^ *V*_2_ chain graphs generated by Day & Hawthorne (2022 ▸). Day *et al.* (2024 ▸) used *Graph* *T*–*T* to examine why some embeddings can occur and others cannot occur, *i.e.* what controls possible topologies for chain structures. To do this, graphs must be generated independently of Euclidean space (and without symmetry constraints). This is a notable difference from the vector method for quotient-graph description and generation of periodic nets (Chung *et al.*, 1984 ▸) and the geometric analysis of nets generated from isomorphic quotient graphs (Eon, 1998 ▸, 1999 ▸), where nets are generated and described using geometric aspects of the graphs (*i.e.* vertices described using metric indices). For the reasons described above, Day & Hawthorne (2022 ▸) developed a new method for the generation of 1-periodic graphs and here we introduce the program *Graph* *T*–*T* for embedding such graphs in Euclidean space.

The software program *GraphTea* (Rostami *et al.*, 2014*a* ▸,*b* ▸) was written as an educational tool for introductory graph theory, and has a visualization routine to aid students in understanding graphs (*e.g.* vertex degree, looped and/or directed edges *etc*.). Using *GraphTea*, the user may draw and manipulate graphs in 2D by changing the relative positions of vertices and the lengths of edges. However, the *GraphTea* visualization interface was designed to facilitate visual comprehension of graphs whereas we are interested in embedding graphs in Euclidean space. Using *GraphTea*, one cannot embed graphs in 2D or 3D Euclidean space while restraining their metric properties. *Graph* *T*–*T* was developed from *GraphTea* to incorporate these capabilities and was used extensively by Day *et al.* (2024 ▸) as described above.

### 1.1. Terminology

Following Day *et al.* (2024 ▸) we define the following terms:

*Chain:* an arrangement of (*T*O_4_)^*n*−^ tetrahedra that (1) links together infinitely in a single direction, (2) has periodic (translational) symmetry, and (3) can be broken into two parts by eliminating a single linkage between adjacent tetrahedra.

*Ribbon:* an arrangement of (*T*O_4_)^*n*−^ tetrahedra that (1) links together infinitely in a single direction, (2) has periodic (translational) symmetry, and (3) cannot be broken into two parts by eliminating a single linkage between adjacent tetrahedra.

*Graph:* a graph, *G* = (*V*, *E*), consists of a set of vertices (*V*) and a set of unordered pairs of vertices called edges (*E*).

*Chain graph:* a 1-periodic graphical representation of a chain of (*T*O_4_)^*n*−^ tetrahedra in which tetrahedra and the linkages between them are represented as vertices and edges, respectively. A chain graph contains only the topological information of the corresponding chain of (*T*O_4_)^*n*−^ tetrahedra and does not contain any geometrical information.

*Geometric graph:* a geometric graph is a graph that is defined at least partly by geometric means. A common definition describes a geometric graph as a graph with straight edges occurring in the Euclidean plane. However, for our purposes, we will define a geometric graph as *a graph with straight edges occurring in Euclidean space*.

*Unit-distance graph:* a geometric graph with all edges of unit length; here, we will generalize this definition slightly: all edges will be of equal length. Once a chain graph has been embedded in Euclidean space, it is transformed into a geometric graph; if any graph is embedded with the constraint of equal edges, it is a unit-distance graph. It follows that a geometric graph or a unit-distance graph is an embedding of a graph or chain graph.

## 2. Rationale for embedding graphs in 3D Euclidean space

**Figure 1:**
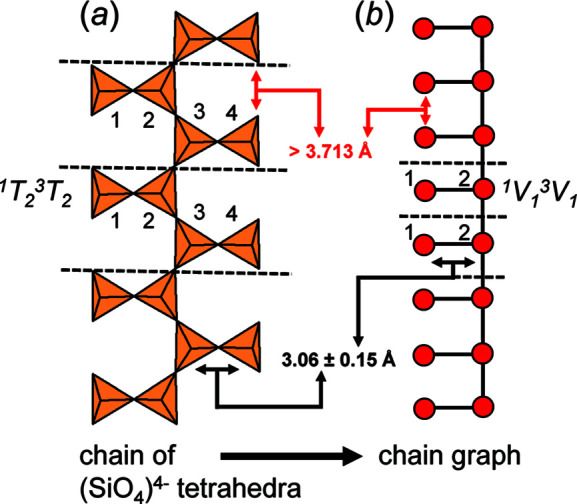
(*a*) The chain of (SiO_4_)^4−^ tetrahedra in astrophyllite-supergroup minerals, and (*b*) the graphical representation of this chain (chain graph) in which tetrahedra are represented as vertices and the linkages between tetrahedra are represented as edges. Red arrows show *T*–*T* separations which are constrained to be at least 3.713 Å during embedding. Black arrows indicate *T*–*T* distances which are constrained to be 3.06±0.15 Å during embedding.

Day & Hawthorne (2022 ▸) and Day *et al.* (2024 ▸) examined topological properties of crystal structures that affect the stability and abundance of the mineral in which they occur. Day & Hawthorne (2020 ▸) described chains of (*T*O_4_)^*n*−^ tetrahedra observed in chain-silicate minerals (inosilicates) where *T* = Si^4+^ plus P^5+^, V^5+^, As^5+^, Al^3+^, Fe^3+^, B^3+^, Fe^2+/3+^, Be^2+^, Zn^2+^ and Mg^2+^, and compared the topology of chains of (*T*O_4_)^*n*−^ tetrahedra using graphs (*chain graphs*) in which tetrahedra are represented by vertices and linkages between tetrahedra are represented by edges, shown in Fig. 1 ▸ for the chain of tetrahedra in the astrophyllite-supergroup minerals (Sokolova *et al.*, 2017 ▸). They classified and compared observed chain arrangements using the expressions *^c^T_r_* and *^c^V_r_*, where *T* denotes tetrahedra, *V* denotes vertices and *r* is the number of tetrahedra (or vertices) with connectivity *c* (from 1 to 4) in the *repeat unit* (unit cell) of the chain of tetrahedra or chain graph. The repeat unit is the part of a chain that can be repeated by translational symmetry operators to produce the complete (quasi-) infinite chain. In Fig. 1 ▸, dashed lines outline the repeat unit of the chain of tetrahedra and the corresponding chain graph. Following Day & Hawthorne (2020 ▸, 2022 ▸) and Day *et al.* (2024 ▸), the connectivity of the chain of tetrahedra in Fig. 1 ▸(*a*) is ^1^ *T*_2_^3^ *T*_2_ and the connectivity of the corresponding chain graph is ^1^ *V*_1_^3^ *V*_1_ [Fig. 1 ▸(*b*)]. The tetrahedra in the repeat unit of the chain are labelled [Fig. 1 ▸(*a*)]. Vertices in the repeat unit of the chain graph are also labelled [(Fig. 1 ▸(*b*)] and the isomorphism relations may be derived using the characteristic polynomial equations of the graph and its subgraphs as described by Day & Hawthorne (2022 ▸).

Day & Hawthorne (2020 ▸) described topological properties common in silicate minerals that are relatively abundant and others that are rare (*e.g.* 4-connected tetrahedra). Day & Hawthorne (2020 ▸, 2022 ▸) showed why some chain arrangements with particular vertex connectivities are not topologically possible and that chain arrangements with stoichiometry *T*O_2.5_–*T*O_2.0_ are not observed in minerals or related synthetic compounds despite being topologically possible. To better understand these observations, they generated all possible non-isomorphic chain graphs for vertex connectivities of 1 to 4. Day *et al.* (2024 ▸) developed a method for testing the compatibility of these chain graphs with the average metrics of chains of tetrahedra occurring in silicates. To implement this, it was necessary to develop software in which each chain graph could be embedded in 3D Euclidean space while restraining metric properties, specifically the distance between linked vertices (*T*–*T* distances) and unlinked vertices (*T*⋯*T* separations).

### 2.1. Restraining metric properties of unit-distance graphs during embedding

To embed chain graphs in Euclidean space while restraining *T*–*T* distances and *T*⋯*T* separations, one must impose net attractive and repulsive forces on the vertices to act as these restraints. One is tempted to think of these restraints as real forces between atoms in the structure (similar to a molecular-mechanics calculation), but this is not the case. The forces in the embedding process are not interatomic forces and we are not self-consistently minimizing some energy function. The ‘forces’ involved in the restraint process are designed to move the vertices of a unit-distance graph towards an ‘optimum’ geometrical arrangement rather than minimize the energy of the overall arrangement (although the process may do this in a crude mean-field type of way). This is done by restraining *T*–*T* distances and *T*⋯*T* separations to values observed in chains of tetrahedra in minerals (Fig. 1 ▸).

Day *et al.* (2024 ▸) calculated the average *T*–*T* distance and *T*⋯*T* separations for all chain-silicate minerals; *T*–*T* distances range from 2.616 to 3.450 Å with an average value of 3.060±0.15 Å. Approximately 94% of the *T*–*T* distances are in the range 2.910–3.210 Å, and values outside this range tend to involve other tetrahedrally coordinated cations. Thus, when embedding chain graphs, we restrain *T*–*T* distances to 3.060±0.15 Å (*i.e.* with an allowed *T*–*T* variance of 5%). The minimum *T*⋯*T* distance is 3.54 Å [Si–Si in marsturite (Kolitsch, 2008 ▸)]; there are no data between 3.540 and 3.713 Å and only ∼20 data points between 3.713 and 3.904 Å. Hence, when embedding chain graphs, we set the minimum *T*⋯*T* separation γ = 3.713 Å. For a particular embedding, the minimum difference allowed between *T*–*T* and *T*⋯*T* is 3.713 − 3.210 = 0.503 Å. Any chain graph that requires *T*–*T* distances smaller or larger than 3.060±0.15 Å and/or *T*⋯*T* separations smaller than 3.713 Å, once embedded in Euclidean space, is unlikely to occur in crystal structures.

#### 2.1.1. Chain arrangements with *T* cations other than Si^4+^

The *T*–*T* distance and *T*⋯*T* restraints (as described in Section 2.1) are based on the average observed *T*–O–*T* distances and *T*⋯*T* separations, where *T* = Si^4+^. Although most chain types contain only (SiO_4_)^4−^ tetrahedra, many groups of chains contain *T* cations other than Si^4+^, such as the sapphirine-supergroup minerals which contain chains of tetrahedra where *T* = Si^4+^, Al^3+^, Fe^3+^, B^3+^ and Be^2+^. Consider the sapphirine-supergroup (Grew *et al.*, 2008 ▸) (rhönite-group) aluminate minerals warkite, Ca_2_Sc_6_O_2_(Al_6_O_18_) (Ma *et al.*, 2015 ▸), and addibischoffite, Ca_2_Al_6_O_2_(Al_6_O_18_) (Ma *et al.*, 2017 ▸), which contain (Al_6_O_18_)^18−^ chains (Day & Hawthorne, 2020 ▸). Here the average *T*–*T* (Al–Al) distance is 3.128 Å as 〈^[4]^Al^3+^–O^2–^〉 = 1.746 Å and 〈^[4]^Si^4+^–O^2–^〉 = 1.625 Å (Gagné & Hawthorne, 2018*a* ▸). Thus, if one wishes to embed (observed or theoretical) chain graphs where vertices represent (AlO_4_)^5−^ tetrahedra rather than (SiO_4_)^4−^ tetrahedra, the metric properties of such chain graphs must be restrained to values in accord with the average Al–O–Al distances and Al–Al separations observed in minerals and related synthetic compounds. This can be done for most of the commonly observed *T* cations using the *T*–O and 〈*T*–O〉 bond lengths given by Gagné & Hawthorne (2016 ▸, 2018*a* ▸,*b* ▸, 2020 ▸) which in some cases show significant differences between different cations, *e.g.* 〈^[4]^B^3+^–O^2−^〉 = 1.475 Å and 〈^[4]^Mg^2+^–O^2−^〉 = 1.939 Å.

## 3. *Graph* *T*–*T* embedding algorithms

### 3.1. The embedding process

The following is a description of how the *T*–*T* and *T*⋯*T* restraints are integrated into the 3D embedding algorithm.

(i) **T*–*T* distances:* the distances between linked vertices are restrained by treating edges as notional springs that behave according to Hooke’s law:

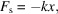

where *F*_s_ is the spring force, *k* is the spring coefficient (stiffness) and *x* is the amount of spring displacement from the equilibrium spring length. Here, the equilibrium spring length represents the ideal *T*–*T* distance in chains of tetrahedra (3.06±0.15 Å). Increasing spring displacement requires increasing *F*_s_ due to repulsion between unlinked vertices.

(ii) **T*⋯*T* separations:* the distances between unlinked vertices are restrained by a mutual repulsive interaction between them described by Coulomb’s law,

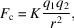

where *F*_c_ is the Coulomb force, *K* is Coulomb’s constant, *q*_1_ and *q*_2_ are the charges associated with each vertex *T*, and *r* is the distance between vertices (charges). As all vertices represent [SiO_4_]^4−^ tetrahedra, all charges are identical and are simply set to 1. Coulomb’s constant, *K*, is adjusted to increase or decrease *F*_c_.

The net notional forces acting on all vertices and the resultant coordinates (*x*, *y* and *z*) of each vertex may be calculated. In this calculation, *F*_s_ may be scaled differently than *F*_c_ to allow embedding of any chain graph even if *T*–*T* and *T*⋯*T* restraints cannot be satisfied exactly. For such cases, *T*⋯*T* separations are forced to be unrealistic (less than 1.16 times the *T*–*T* distance) such that one can identify specific chain topologies that are not compatible with the observed metrics of chains of tetrahedra.

The cost function of the optimization of the embedding process involves (1) minimizing the difference between the nominal *T*–*T* distance and the calculated separation of linked vertices in the graph, and (2) minimizing the difference between the nominal minimum distance between unlinked vertices and the calculated separation of linked vertices in the graph where the difference is positive and giving the difference zero weight where it is negative.

### 3.2. Embedding software: *Graph* *T*–*T*

*Graph* *T*–*T* (V1.0Beta) is a web-based visualization software for embedding graphs in 3D Euclidean space while restraining the metric properties of the resultant unit-distance graph. The metric properties of unit-distance graphs are computed using a 3D spring-force algorithm in which the initial spring length (*T*–*T* distance) can be set to any value (*e.g.* 〈*T*–*T*〉 = 3.06±0.15 Å). This algorithm was constructed using the open-source algorithms *3d-force-graph* and *ngraph* (Appendix *A* [App appa]). A third open-source algorithm, *d3-force* (Appendix *A* [App appa]), was used which utilizes a Verlet integration (Verlet, 1967 ▸), as would be typically applied to an *n*-body problem to integrate Newton’s equations of motion, where vertices represent bodies with mass equal to one (all vertices represent the same *T* cation, *e.g.* Si^4+^).

The net forces acting on vertices of a particular unit-distance graph are calculated iteratively according to Newton’s laws of motion using a Barnes–Hut (*n*-body) simulation (Barnes & Hut, 1986 ▸). For *F*_s_ calculations, *k* is adjustable to allow deviation from the set spring length. For *F*_c_ calculations, *K* is adjustable to allow the occurrence of *T*⋯*T* separations less than the threshold value of γ = 3.713 Å (less than 1.16 times the *T*–*T* distance). The *F*_c_ calculation involves all unique pairs of vertices, and the run-time (*r_t_*) of the *Graph* *T*–*T* algorithm increases exponentially as the number of vertices (*n*) in the input graph increases (*r_t_* increases proportionally to *n* ^2^). Thus, *r_t_* is impractically large for chain graphs with many vertices. To avoid this problem, the Barnes–Hut simulation groups adjacent vertices as *bodies* and calculates the position of the centre of charge of that body. The net repulsive force exerted on all other bodies from the centre of charge of each body is calculated and used to calculate a new position for each vertex after each iteration. Vertices are grouped using a quadtree structure where the chain is divided into *q_c_* cells that contain *n_qc_* vertices. Averaging the position of *n_qc_* vertices introduces a small amount of error in the *F*_c_ calculation and the resultant vertex coordinates. However, *q_c_* may be adjusted, and as *q_c_* approaches *n*, *n_qc_* decreases and the error in *F*_c_ decreases. If *q_c_* = *n*, then *n_qc_* = 1 and the Barnes–Hut simulation is no different from a brute-force algorithm where *F*_c_ is calculated for all unique pairs of vertices. Of course, by setting *q_c_* = *n*, final vertex coordinates will have less positional error than when *q_c_* < *n* but will result in a value of *r_t_* that is impractically large. These positional errors are typically negligible with respect to the allowed *T*–*T* variation.

The *embedding parameters* in *Graph* *T*–*T* include spring coefficient (*k*), Coulomb’s constant (*K*), spring length, drag coefficient, theta, time step and cooldown time, all of which may be adjusted by the user. The drag coefficient can be increased to damp the movement of vertices after each iteration to decrease oscillation and speed up convergence. Theta is adjusted to increase or decrease *q_c_* in the Barnes–Hut simulation. The time step is adjusted to increase or decrease the speed of each iteration and controls how the discretization of each equation of motion is performed. The cooldown time is adjusted to increase or decrease the number of iterations before the calculation stops. Unit-distance graphs that contain a repeat unit with a large number of vertices often require a large number of iterations before convergence is reached and thus require a relatively long cooldown time.

Non-isomorphic chain graphs may require slightly different embedding parameters to produce unit-distance graphs that satisfy the *T*–*T* and *T*⋯*T* restraints. Furthermore, a single chain graph may be embedded using different parameters to produce geometrically different unit-distance graphs that satisfy the *T*–*T* and *T*⋯*T* restraints. Thus Day *et al.* (2024 ▸) determined the ideal ranges for each parameter by embedding a series of chain graphs using *Graph* *T*–*T* until the minimum *T*⋯*T* separations were maximized, and the average *T*–*T* distances were in best agreement with the equilibrium spring length. These parameters are listed by Day *et al.* (2024 ▸, Tables 1–4). Some unit-distance graphs show distortion that occurs over many repeat units [*e.g.* modulated and helical chains (Day *et al.*, 2024 ▸)] and thus must be input into *Graph* *T*–*T* by multiplying *n_t_* (number of vertices in the repeat unit) by a variable *p*, where *p* = 9–50 for most chain graphs. For example, a ^2^ *V*_2_^3^ *V*_2_ chain graph with *p* = 9 corresponds to a chain graph with *n* vertices where *n* = ∑*r* × *p* = 4 × 9 = 36 vertices.

As described in Section 2.1, the average distance between tetrahedra (*T*–*T* distances) observed in chain-silicate minerals is 3.06 Å, and thus setting the equilibrium spring length in *Graph* *T*–*T* to 3.06 Å may seem practical. However, using such a small spring length results in unit-distance graphs that are visually difficult to comprehend as vertices appear very close together. To overcome this problem, we set the spring length to 50.00 to ensure that the geometry of unit-distance graphs produced with *Graph* *T*–*T* is easily understood. Although this increases the convergence time (minimum cooldown time) for some chain graphs, it does not affect the *Graph* *T*–*T* outputs (geometry of unit-distance graphs) as *F*_s_ and *F*_c_ and the resultant *T*–*T* distances and *T*⋯*T* separations are simply scaled by a factor of 16.34 (3.06 × 16.34 = 50.00 Å and γ = 50 × 1.16 = 58 Å). After the embedding process is complete, they may be re-scaled (*e.g.* 50/16.34 = 3.06 Å and 58/16.34 = 3.55 Å) such that they can be compared with *T*–*T* distances and *T*⋯*T* separations observed in crystal structures.

#### 3.2.1. Convergence of the embedding process

In *Graph* *T*–*T*, when the first phase of the embedding process starts, all vertices occupy the same position (*x*, *y*, *z* = 0, 0, 0) in the 3D visual rendering space and thus all *T*–*T* distances and *T*⋯*T* separations are zero. As embedding progresses, *T*–*T* distances will approach the set spring length and *T*⋯*T* separations will increase due to repulsion involving *F*_c_. The average *T*⋯*T* separation will increase until *T*–*T* distances reach a point (restrained by *k*, the spring coefficient) such that *F*_s_ is no longer compatible with further increase in the *T*⋯*T* separations. At this point, the unit-distance graph has *converged*.

The minimum, maximum and average *T*–*T* distances and *T*⋯*T* separations are calculated and reported in real time such that the user can determine when a particular unit-distance graph has converged. Once convergence is reached, vertices will continue to respond to *F*_s_ and *F*_c_ and each vertex will oscillate around a point in response to these forces. Before convergence is reached, reported *T*–*T* distances and *T*⋯*T* separations will trend towards higher or lower values although the rate at which this occurs may be relatively slow depending on *k*, *K* and the drag coefficient. For unit-distance graphs that have converged using large values of *k* and *K*, the degree of vertex oscillation may be relatively large, and convergence will never result in a single set of coordinates for any given vertex, but rather a range of coordinates. Therefore, average *T*–*T* distances and minimum *T*⋯*T* separations are reported as ranges [R〈*T*–*T*〉 and R〈*T*⋯*T*〉_min_ in Tables 1–4 of Day *et al.* (2024 ▸)] and are referred to as the *compatibility parameters* of the unit-distance graph to which they correspond. Note that *T*⋯*T* is always reported as a minimum and that, for some unit-distance graphs, variation due to oscillation in *T*–*T* and *T*⋯*T* is negligible (when *k* and *K* are relatively small) and such values are reported as single integers (denoted as 〈*T*–*T*〉 and *T*⋯*T*_min_) rather than ranges as described above.

A chain graph which has been embedded using *Graph* *T*–*T* to produce a unit-distance graph that has converged can be described as:

(i) *Compatible*: if a unit-distance graph converges such that the *T*–*T* and *T*⋯*T* restraints are satisfied, the corresponding chain graph may correspond to a chain of tetrahedra and is considered as potentially *compatible* with a crystal structure.

(ii) *Incompatible*: if a unit-distance graph converges such that the *T*–*T* and/or *T*⋯*T* restraints are not satisfied, the corresponding chain graph cannot correspond to a chain of tetrahedra and is considered *incompatible* with a crystal structure. If a unit-distance graph does not converge (Section 4.3), it is incompatible.

#### 3.2.2. Two-step embedding: a solution for metastable unit-distance graphs

As the embedding process progresses and convergence is achieved, any given vertex should occupy a position with associated *T*–*T* distances that are as close as possible to the equilibrium spring length (*e.g.* 3.06±0.15 Å) and *T*⋯*T* separations that are as large as possible. For some unit-distance graphs, particularly complicated ones with a high average vertex connectivity, this is not the case as one or more vertices may occupy a non-ideal position (a *false minimum*) with respect to the associated *T*–*T* distances and *T*⋯*T* separations. Any unit-distance graph that contains one or more vertices that occupy a false minimum is referred to as *metastable*. Metastable unit-distance graphs occur where one or more vertices become *trapped* in a position where a temporary increase in the corresponding *T*–*T* distances and *T*⋯*T* separations (*F*_s_ and *F*_c_) towards less ideal values is required for that vertex to move to a more ideal position with respect to the ideal *T*–*T* distances and *T*⋯*T* separations. Metastable unit-distance graphs will converge to false minima and will show large discrepancies between the minimum and maximum *T*–*T* distances and *T*–*T* separations significantly smaller than the threshold values.

To reduce the probability of generating metastable unit-distance graphs, graphs are embedded using a two-phase procedure. In the first phase, the spring coefficient (*k*) is relatively small (*k* = 0.0008) for the first 15 s of the embedding process to allow vertices to move rapidly to positions that are close to ideal. In this phase, the probability that vertices become trapped at false minima is low as *F*_s_ is smaller than the recommended user-defined embedding parameters and more easily counteracted by *F*_c_. In the second phase of embedding, the user-defined embedding parameters are applied to the unit-distance graph, the positions of vertices are refined and the minimum and maximum *T*–*T* distances and minimum *T*⋯*T* separations are reported by *Graph* *T*–*T*. During the first phase of embedding, *T*⋯*T* separations are often significantly smaller than during the second phase as *F*_c_ is a function of the distance between unlinked vertices (*r* ^2^, Section 3.1), and at a particular distance between any two unlinked vertices (∼7.43 Å = 2 × 3.713 Å), *F*_c_ becomes negligible.

**Figure 2:**
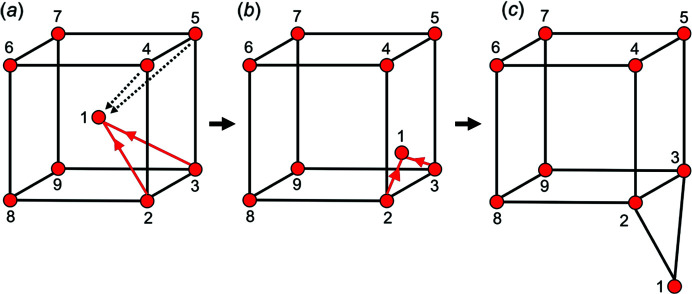
(*a*) An example of a metastable unit-distance graph in which vertex 1 occupies a *false-minimum* position where the 1–2 and 1–3 edges are shorter than the other (black) edges and thus vertex 1 experiences *F*_s_ in the direction of the red arrows. In response to *F*_s_, vertex 1 also experiences *F*_c_ (black dashed arrows) as the distance between vertex 1 and all other vertices to which it is not linked is shorter than the length of the black edge. For vertex 1 to move from this false minimum, *F*_s_ must increase temporarily as shown in (*b*) in order to converge to the ideal position shown in (*c*).

Consider the cubic unit-distance graph in Fig. 2 ▸(*a*); black edges are 3.06 Å long, vertex 1 occupies a position in the centre of the cube and is linked to vertices 2 and 3 by the red edges. The length (*L*) of the red 1–2 and 1–3 edges is ( 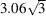 )/2 = 2.65 Å and is shorter than the minimum allowed *T*–*T* distance of 2.91 Å and therefore is not allowed. During embedding, vertex 1 will be subject to a spring force in the direction of the red arrows to increase the 1–2 and 1–3 edge lengths. However, in doing so, the *T*–*T* separation distance between vertex 1 and vertices 6, 7, 8 and 9 will decrease and the repulsion force on vertex 1 will increase. Regardless of which face of the cube vertex 1 moves towards, the counteracting effects of *F*_s_ and *F*_c_ will prevent the vertex from moving to a position in which both the *T*–*T* distance and *T*⋯*T* separation restraints are satisfied; thus the unit-distance graph in Fig. 2 ▸(*a*) is metastable. For this graph to move away from the metastable state and perhaps converge to a stable state, vertex 1 must move past vertices 2 and 3 and undergo a temporary increase in *F*_s_ from subsequent shortening of the 1–2 and 1–3 edges [Fig. 2 ▸(*b*)]. The size of *F*_s_ and *F*_c_ will allow this type of movement in the first phase of embedding but not in the second phase. At the end of phase one, vertex 1 has moved to a position in which the *T*–*T* distance and *T*⋯*T* separation distance are much closer to the ideal values [Fig. 2 ▸(*c*)]. The position of vertex 1 [Fig. 2 ▸(*c*)] is then refined during the second phase of embedding.

## 4. Examples: embedding chain graphs using *Graph* *T*–*T*

### 4.1. The amphibole ribbon

**Figure 3:**
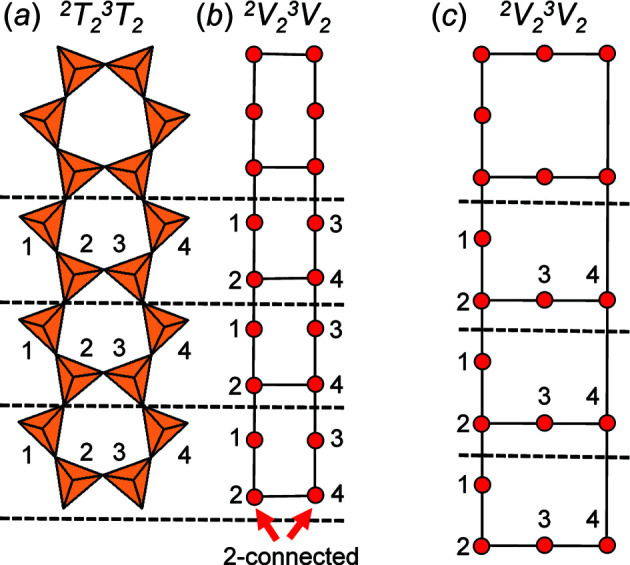
(*a*) The ^2^ *T*_2_^3^ *T*_2_ chain of (SiO_4_)^4−^ tetrahedra in amphibole-supergroup minerals with a repeat unit that contains four tetrahedra; (*b*) the corresponding ^2^ *V*_2_^3^ *V*_2_ chain graph with a repeat unit that contains four vertices; (*c*) another ^2^ *V*_2_^3^ *V*_2_ chain graph that is non-isomorphic (topologically different) with the chain graph in (*b*).

Amphibole-supergroup minerals (Hawthorne *et al.*, 2012 ▸) comprise the largest group of chain-silicate minerals and contain ^2^ *T*_2_^3^ *T*_2_ ribbons of (*T*O_4_)^*n*−^ tetrahedra [Fig. 3 ▸(*a*)]; the corresponding ^2^ *V*_2_^3^ *V*_2_ chain graph is shown in Fig. 3 ▸(*b*). To determine to what degree the topological properties of this chain arrangement contribute to the relatively high stability and abundance of amphiboles, one must compare the compatibility parameters (given by *Graph* *T*–*T*) of this arrangement with other topologically similar arrangements. To do this, Day & Hawthorne (2022 ▸) generated all possible, non-isomorphic ribbons with identical vertex connectivity ^2^ *V*_2_^3^ *V*_2_; one of these ribbons is shown in Fig. 3 ▸(*c*).

**Figure 4:**
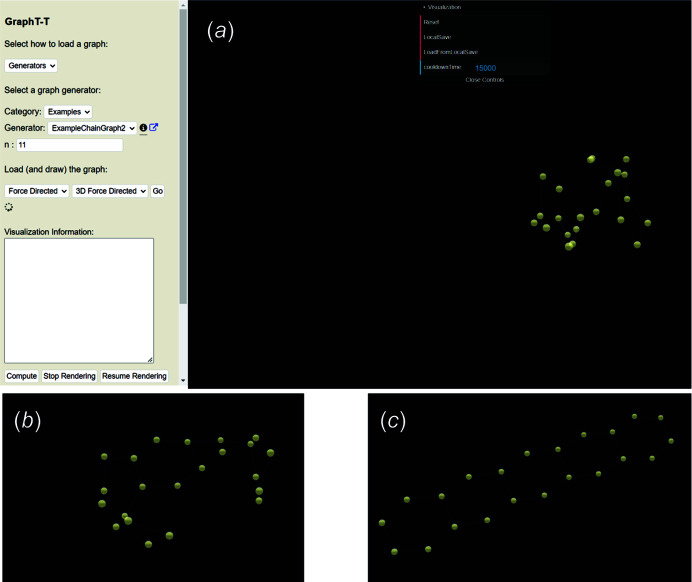
(*a*) The *Graph* *T*–*T* interface showing the first few seconds of the first phase of the embedding process for a ^2^ *V*_2_^3^ *V*_2_ unit-distance graph. (*b*) The unit-distance graph after 2–5 s showing rapid expansion and movement of vertices towards ideal positions with respect to the ideal *T*–*T* distances and *T*⋯*T* separations. (*c*) The unit-distance graph after 10–15 s where vertices occupy positions close to ideal with respect to the *T*–*T* and *T*⋯*T* constraints.

Several chain graphs have been included in *Graph* *T*–*T* as examples to allow the user to test sets of parameters on a series of non-isomorphic graphs with different vertex connectivities. The chain graph in Fig. 3 ▸(*b*) corresponds to *ChainGraph2*; this chain graph can be loaded using the *Generator* dropdown menu and the length of this chain graph can be adjusted by setting the parameter *n* as shown in Fig. 4 ▸(*a*), where *n* is the number of repeat units included in the visual rendering. As discussed in Section 3.2.2, in the first phase of the embedding process, vertices occupy the same position and, after ∼1 s, vertices will appear clustered [Fig. 4 ▸(*a*)] as they begin to move away from one another [Fig. 4 ▸(*b*)] to assume positions in which the *T*–*T* distance and *T*⋯*T* separation restraints are satisfied (or close to satisfied), as shown in Fig. 4 ▸(*c*).

**Figure 5:**
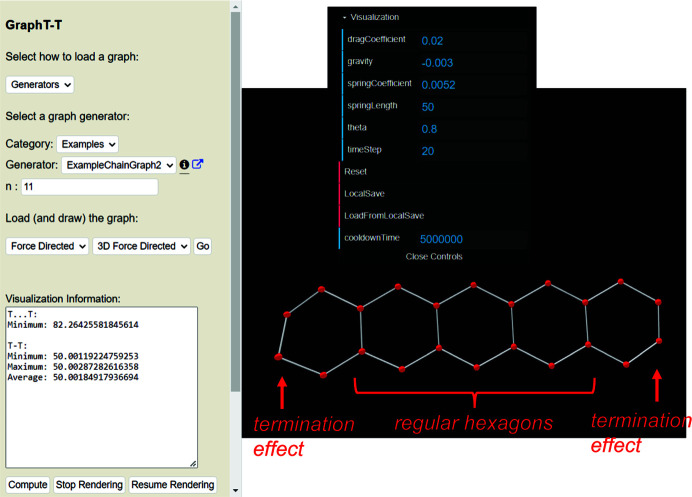
The *Graph* *T*–*T* interface showing the ^2^ *V*_2_^3^ *V*_2_ chain in amphibole-supergroup minerals that has converged to a compatible unit-distance graph using the embedding parameters recommended by Day *et al.* (2024 ▸). Note the asymmetry of the hexagons at each end of the unit-distance graph due to termination effects.

Once the first phase of embedding has finished and the second phase has begun, vertices will change colour from yellow to red and the user-specified embedding parameters will be applied to the unit-distance graph (Fig. 5 ▸). The unit-distance graph in Fig. 5 ▸ is produced once the cooldown time for the second phase of embedding has elapsed. Here, R〈*T*–*T*〉 = 50.001–50.003 Å is in excellent agreement with the set equilibrium spring length (50) and R〈*T*⋯*T*〉_min_ = 81.493–82.863 Å which is significantly larger than γ = 58 Å, and thus we confirm that this graph is *compatible*.

**Figure 6:**
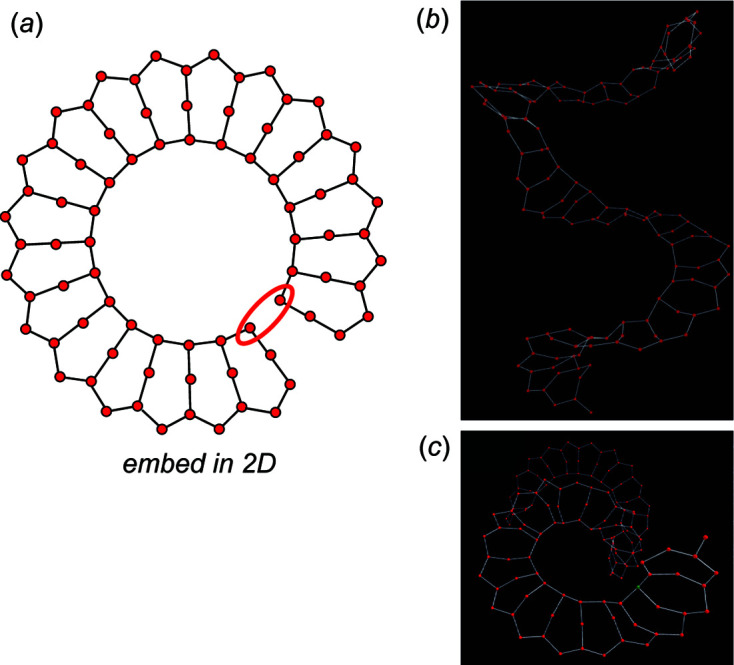
(*a*) The unit-distance graph produced by embedding the chain graph shown in Fig. 3 ▸(*c*) in 2D. This chain is forced to curve in on itself to ensure approximately equal *T*–*T* distances. At a particular length (number of tetrahedra, *n*), this results in *T*⋯*T* separations that are too short (shown with a red ellipse). This unit-distance graph embedded in 3D viewed (*b*) along the long axis of the chain and (*c*) into the long axis of the chain. Note how the chain is forced to form a helical arrangement to prevent unrealistically short *T*⋯*T* separations as shown with the red ellipse in (*a*).

Embedding the chain graph in Fig. 3 ▸(*c*) in 2D results in the unit-distance graph in Fig. 6 ▸(*a*). In 2D, this unit-distance graph is forced to curve to shorten the 4–4 edge [Fig. 3 ▸(*c*)] to make all edges of equal length. However, at a particular value of *n*, the chain is forced to curve in on itself, resulting in unrealistically small *T*⋯*T* separations, *e.g.* the 4–4 separation, shown by a red ellipse, is approximately the same size as the *T*–*T* distances [Fig. 6 ▸(*a*)]. Thus, we conclude that this chain graph is incompatible in 2D. Embedding the chain graph in Fig. 3 ▸(*c*) in 3D using *Graph* *T*–*T* results in the unit-distance graphs in Figs. 6 ▸(*b*) and 6 ▸(*c*). This unit-distance graph is forced to form a helix in order to equalize edge lengths and prevent unrealistic *T*–*T* separations (*e.g.* the 4–4 separation). Here, R〈*T*–*T*〉 = 48.146–51.956 Å which is in good accord with the set equilibrium spring length (50 Å) and R〈*T*⋯*T*〉 = 61.870–63.607 Å which is larger than γ = 58 Å; thus, we confirm this chain graph is *compatible*. This type of geometric distortion is referred to as medium-range modification by Day *et al.* (2024 ▸) and is discussed in more detail in Section 4.2.1. A more detailed analysis of the compatibility parameters for the unit-distance graph shown in Fig. 5 ▸ and other chains with vertex connectivity ^2^ *V*_2_^3^ *V*_2_ (*e.g.* Fig. 6 ▸) is given by Day *et al.* (2024 ▸).

The embedding parameters used for the chain graphs in Figs. 3 ▸(*b*) and 3 ▸(*c*) were taken from Day *et al.* (2024 ▸). These parameters were determined experimentally and refined using a method described in Section 4.3.

### 4.2. Termination effects

As all chains of tetrahedra, and the corresponding chain graphs, are 1-periodic, one must select some finite length (number of repeat units) of the chain to embed using *Graph* *T*–*T*. Vertices at either end of a finite segment of any chain graph will be subject to different net forces during embedding compared with analogous vertices (of a different repeat unit) that occur at, or close to, the middle of the chain. This is because the net forces acting on a given vertex are affected by the connectivity of that vertex, the number of unlinked vertices to which it is adjacent, and its proximity to each of those unlinked vertices. Therefore, vertices (and edges) at the end of any unit-distance graph will converge to a geometry different from that of the middle of the unit-distance graph. For example, consider the chain graph in Fig. 3 ▸(*b*). Each repeat unit contains two 2-connected vertices (vertices 1 and 3) and two 3-connected vertices (vertices 2 and 4) except for the repeat units at both ends of the chain graph. Vertices 2 and 4 at the end of the chain graph [shown with red arrows, Fig. 3 ▸(*b*)] are 2-connected rather than 3-connected, and thus are subject to a different net force during embedding compared with the translationally symmetric vertices 2 and 4 in the other repeat units. Embedding this chain graph results in a chain geometry at both ends of the corresponding unit-distance graph that is different from the rest of the chain. This is shown in Fig. 5 ▸, where the middle of the unit-distance graph consists of three regular, edge-sharing hexagons in which all vertices lie on a single plane. The hexagons on either end have different geometries and contain vertices that lie out of the plane containing the middle hexagons; this is a called a *termination effect*.

#### 4.2.1. Recommended *n* for input chain graphs

Vertices that experience termination effects always have a lower connectivity than equivalent (translationally symmetric) vertices and thus have more freedom to move in response to *F*_s_ and *F*_c_. It follows that termination effects tend to skew the average *T*–*T* distances and *T*⋯*T* separations to larger values. For chain graphs in which ∑*r* (*^c^V_r_*) is relatively small (1–8) and where *n* (the number of repeat units) is relatively small (1–4), termination effects may increase the *T*–*T* distances and *T*⋯*T* separations to the point where the user may erroneously identify a unit-distance graph as *compatible* when in fact it is *incompatible* or *vice versa* (see Section 3.2.1). This problem is easily mitigated by using *n* ≥ 5 for any chain graph that the user wishes to embed. Even for chain graphs with ∑*r* = 1–8, setting *n* ≥ 5 results in an increase in *T*–*T* and *T*⋯*T* (in the corresponding unit-distance graph) due to termination effects that is negligible with respect to the positional errors of vertices once convergence is reached. In general, as *n* increases, the ratio of vertices subject to end effects and those not subject to end effects (vertices in the middle of the chain) decreases and thus any increase in *T*–*T* and *T*⋯*T* due to end effects is inversely correlated with *n*. Therefore, we recommend that one sets *n* as large as possible for any chain graph one wishes to embed using *Graph* *T*–*T*. However, one must also consider realistic computation times and the increase in probability of a metastable unit-distance occurring with increasing *n*.

Particular chain graphs require different types of geometric distortion [*e.g.* helical arrangements, Figs. 6 ▸(*a*)–6 ▸(*c*)] in order to converge to a compatible unit-distance graph. This type of geometric distortion is referred to as *medium-range modification* by Day *et al.* (2024 ▸), who provide a more detailed analysis of this phenomenon than is given here. Unit-distance graphs that experience medium-range modification often have repeat units that are significantly larger (contain many more vertices) than the corresponding chain graph and thus it is recommended that *n* is set to a large value to ensure that at least one complete repeat unit can be recognized by the user once convergence is reached. This will ensure that values are reported for all symmetrically non-equivalent *T*–*T* distances and *T*⋯*T* separations in the repeat unit and that an accurate analysis of the compatibility of the unit-distance graph can be made.

### 4.3. Refining embedding parameters: the ^4^ *V*_2_ shoelace graph

Assuming a starting point as the default set of embedding parameters (Section 3.2), one may iteratively embed a given chain graph and refine the embedding parameters based on how or if the compatibility parameters (reported *T*–*T* distances and *T*⋯*T* separations) of the resultant unit-distance graph trend towards ideal values (*i.e.* if the average *T*–*T* distance trends towards the set spring length with each embedding iteration).

Reaching an endpoint and determining the exact value of each embedding parameter that results in a unit-distance graph with ideal geometry (*i.e.* *T*–*T* distances as close as possible to the set spring length) can be difficult and time consuming. However, for most graphs, the embedding parameters need not be refined to such a high degree as the principal goal of using *Graph* *T*–*T* is to determine whether a chain graph is compatible or incompatible with the metrics of crystal structures, and this can almost always be determined well before each embedding parameter is fully refined. In the following example, we show how by iteratively refining *k* and *K*, one can determine if a given chain graph is compatible or incompatible.

**Figure 7:**
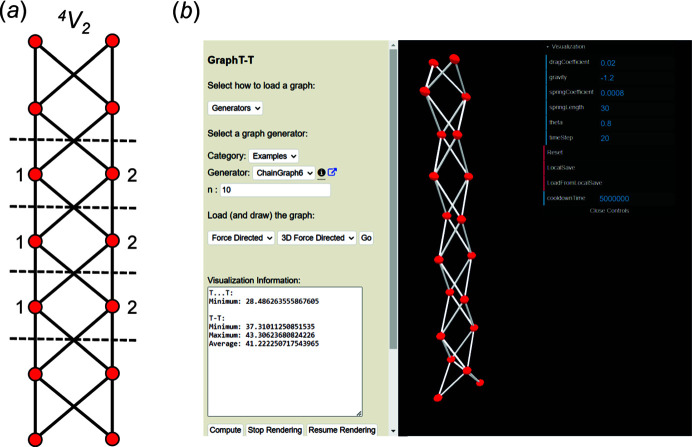
(*a*) The ^4^ *V*_2_ ‘shoelace’ chain graph and (*b*) the corresponding unit-distance graph embedded using the default embedding parameters shown in the visualization menu. Although this graph converges, it is incompatible as R〈*T*–*T*〉 and R〈*T*⋯*T*〉_min_ are significantly larger and smaller than the set spring length (30 Å), respectively.

**Figure 8:**
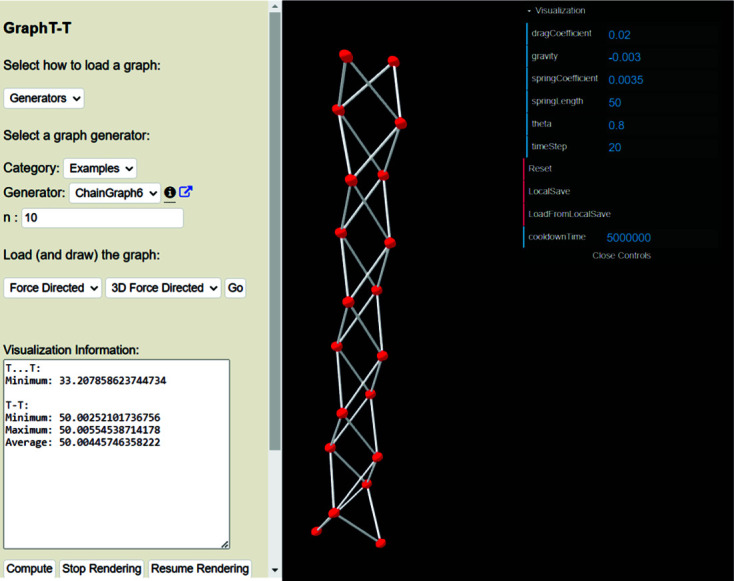
The ^4^ *V*_2_ ‘shoelace’ unit-distance graph embedded using the embedding parameters recommended by Day *et al.* (2024 ▸). This graph converges and has *T*–*T* distances (R〈*T*–*T*〉 = 50.003–50.006 Å) in excellent agreement with the set spring length (50 Å) but is incompatible as *T*⋯*T*_min_ = 33.208 Å.

Consider the chain graph in Fig. 7 ▸(*a*) with vertex connectivity ^4^ *V*_2_ which is referred to by the authors as the *shoelace* graph and is loaded in *Graph* *T*–*T* as example *ChainGraph6*. To determine whether this chain graph is compatible, we begin by embedding it with *Graph* *T*–*T* using the default embedding parameters in Fig. 7 ▸(*b*) and *n* is set to 10 to minimize termination effects. Using these embedding parameters, the chain graph quickly converges to a unit-distance graph with R〈*T*⋯*T*〉_min_ = 28.056–28.541 Å and R〈*T*–*T*〉 = 37.310–43.306 Å and is thus incompatible (using the default embedding parameters) as the set spring length is 30 Å. Next, we can set the embedding parameters to those recommended by Day *et al.* (2024 ▸) as shown in Fig. 8 ▸. Using these parameters, the chain graph converges much more slowly to a unit-distance graph with *T*⋯*T*_min_ = 33.208 Å and R〈*T*–*T*〉 = 50.003–50.006 Å which is thus incompatible (using the embedding parameters shown in Fig. 8 ▸) as *T*⋯*T*_min_ (= 33.208 Å) is less than the set spring length (50 Å). Here, *k* has been increased and *K* has been decreased with respect to the values of *k* and *K* used to embed this graph in Fig. 7 ▸(*b*). As a result, there is less competition between *F*_s_ and *F*_c_ and a negligible degree of vertex oscillation once the unit-distance graph in Fig. 8 ▸ has converged; this is apparent from comparison of the compatibility parameters for Figs. 7 ▸(*a*) and 8 ▸ shown in Table 1 ▸. Next, one may wish to increase *T*⋯*T*_min_ (as reported in Fig. 8 ▸) by increasing *K* and decreasing *k*. These results are shown in Table 1 ▸ (rows [3]–[5]) where *K* is increased from −1.2 to −2.5 to −10.0 while keeping *k* the same (0.001). This results in a progressive increase in *T*⋯*T*_min_ towards the allowed threshold of γ = 58 Å, but also a progressive increase in R〈*T*–*T*〉 away from the set spring length (50 Å). This behaviour is commonly observed for chain graphs with a high average vertex connectivity (*e.g.* ^4^ *V*_2_) and is characteristic of incompatible graphs.

**Figure 9:**
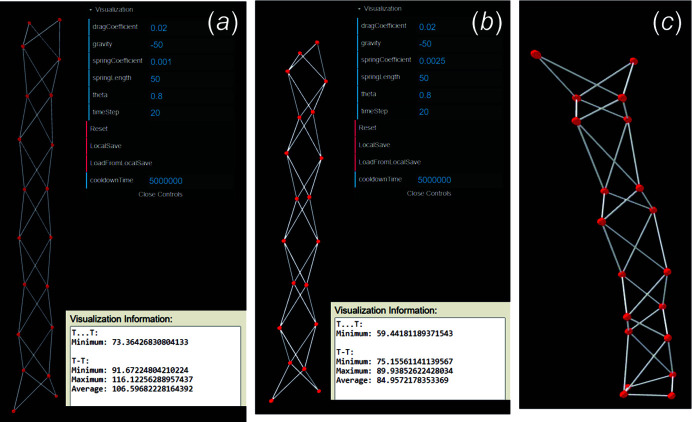
(*a*) The ^4^ *V*_2_ ‘shoelace’ unit-distance graph where R〈*T*–*T*〉 = 91.301–116.635 Å and is thus incompatible where *k* = 0.001 and *K* = −50. In an attempt to decrease R〈*T*–*T*〉, *k* is increased to 0.0025 and the unit-distance graph in (*b*) is produced where R〈*T*–*T*〉 = 74.139–91.431 Å and R〈*T*⋯*T*〉_min_ = 57.597–59.950 Å. Thus, one may conclude this chain graph is incompatible as any further increase in *k*, in an attempt to reduce R〈*T*–*T*〉, will result in a decrease in R〈*T*⋯*T*〉_min_ to values below γ = 58 Å. This is shown in (*c*) where *k* is increased to 0.02 and the resultant unit-distance graph does not converge.

In Fig. 9 ▸(*a*) (Table 1 ▸, row [6]), *K* is increased to −50 and R〈*T*⋯*T*〉_min_ = 72.474–73.803 Å, but to accommodate this change, R〈*T*–*T*〉 also increases and is now approximately double the set spring length. Consequently, one may now attempt to iteratively increase *k* to decrease R〈*T*–*T*〉, and this is shown in Table 1 ▸ (rows [7]–[12]); however, *k* can only be increased to 0.0025 before R〈*T*⋯*T*〉_min_ drops below γ = 58 Å [Fig. 9 ▸(*b*), Table 1 ▸, row [9]]. Any value of *k* ≥ 0.003 where *K* = −50 results in a unit-distance graph that does not converge and thus no values are reported in Table 1 ▸ for [10]–[12]. In Fig. 9 ▸(*c*), the non-convergent unit-distance graph is shown in which all *T*–*T* distances and *T*⋯*T* separations show significant deviation from one another. At this point, based on the results in Table 1 ▸, one can conclude that the ^4^ *V*_2_ ‘shoelace’ graph is incompatible as there is clearly no combination of *k* and *K* in which both the *T*–*T* and *T*⋯*T* restraints are satisfied. Although only two of the embedding parameters (*k* and *K*) were varied for the ^4^ *V*_2_ graph, Day *et al.* (2024 ▸) determined that if a graph cannot be embedded to produce a compatible unit-distance graph by iteratively refining *K* and *k* (as described above), the graph is almost always incompatible irrespective of how the other embedding parameters are set.

Refining the embedding parameters for compatible graphs is much simpler and, for most cases, the compatibility of a graph is immediately apparent if the embedding parameters recommended by Day *et al.* (2024 ▸) are used. Thus, once the compatibility was determined for a graph, embedding parameters were not fully refined by Day *et al.* (2024 ▸) as there was no need to continue the refinement process any further. For example, consider the graph in Fig. 5 ▸: on embedding using the recommended values for each parameter, it is immediately apparent that the graph is compatible as the compatibility parameters are in close accord with the *T*–*T* and *T*⋯*T* restraints. However, it may be possible to improve the compatibility parameters (*i.e.* increase *T*⋯*T*_min_) by further refinement of *k* and *K*. The drag coefficient and/or theta values may also be refined but their effect on the final compatibility parameters is typically minimal.

## 5. Summary

*Graph* *T*–*T* (V1.0Beta) has been developed to embed 1-periodic chain graphs in 3D Euclidean space. It uses a combination of notional spring-force algorithms (see links to open-source code in Appendix *A* [App appa]), and during the embedding process, the distances between unlinked and linked vertices (*i.e.* distance between tetrahedra) of the corresponding unit-distance graph are restrained to be in accord with those in chain-silicate minerals and related synthetic compounds. Here, examples are provided to show how one can iteratively refine the embedding parameters (*e.g.* spring coefficient, *k*, and Coulomb’s coefficient, *K*) to determine if the input chain graph is compatible or incompatible with the observed metrics of chains of *T*O_4_ tetrahedra. Compatible chain graphs may form crystal structures and incompatible chain graphs are unlikely to form crystal structures. For example, *Graph* *T*–*T* has been used extensively by Day *et al.* (2024 ▸) who embedded a suite of observed and hypothetical chain arrangements generated by Day & Hawthorne (2022 ▸) to identify topological properties of chain graphs that control properties such as flexibility (or rigidity). They showed that most chains with stoichiometry *T*O_2.5_–*T*O_2.0_ are relatively rigid and incompatible, which may explain why such chain stoichiometries are not observed in chain-silicate minerals or related synthetic compounds.

### 5.1. Future work

*Graph* *T*–*T* can be used to determine the compatibility of any 0D to 3D arrangement of polyhedra if the allowed *T*–*T* distances and *T*⋯*T* separations observed in analogous crystal structures are determined prior to the embedding process. Currently, we are working on modifying *Graph* *T*–*T* to allow analysis of mixed polymerizations of tetrahedra (*e.g.* aluminosilicate [(Al^3+^,Si^4+^)O_*n*_] chains) where embedding parameters are specified for sets of vertices that correspond to different cations (*e.g.* Si^4+^ and Al^3+^, or Si^4+^ and As^5+^). This will also allow embedding of chain graphs with vertices corresponding to Si^4+^ and O^2−^, providing the opportunity to determine the effect of repulsion between O^2−^ anions on the compatibility of such graphs.

This version of *Graph* *T*–*T* is still undergoing beta testing by the authors; please report any problems or suggestions to the corresponding author. A web-based version of *Graph* *T*–*T* is available at https://graphtt.github.io. The open-source code can be accessed at https://github.com/GraphTT/graphtt.github.io/ where a Zip file can be downloaded containing all component files required to set-up and run *Graph* *T*–*T* locally. This Zip file and installation instructions are also available as supporting information. For information about how to install *Graph* *T*–*T* locally and how to use *Graph* *T*–*T*, refer to Appendix *A* [App appa].

## Supplementary Material

Revised instructions for running GraphT-T locally. DOI: 10.1107/S2053273324002523/uv5025sup1.pdf

New Zip file containing all component files to install the GraphT-T program locally on a user's computer. DOI: 10.1107/S2053273324002523/uv5025sup2.zip

## Figures and Tables

**Table 1:** Compatibility parameters for the ^4^ *V*_2_ shoelace graph for different values of *k* (spring coefficient) and *K* (Coulomb’s constant) R〈*T*–*T*〉 and R〈*T*⋯*T*〉_min_ values that are compatible and incompatible with the set spring length are shown in bold and italic, respectively. Apart from *k* and *K*, default [1] and recommended [2] embedding parameters are identical (see Figs. 7 ▸ and 8 ▸) other than spring length which is set to 30 Å and 50 Å, respectively. For some values of *k* and *K*, variation in *T*–*T* and/or *T*⋯*T* (the degree of vertex oscillation), once converged, is negligible and thus R〈*T*–*T*〉 and R〈*T*⋯*T*〉_min_ are reported as single integers (marked with *) rather than ranges.

Selected *k* and *K*	R〈*T*–*T*〉 (Å)	R〈*T*⋯*T*〉_min_ (Å)	Fig.
[1] *k* = 0.0008, *K* = −1.2 (default embedding parameters)	*37.310–43.306*	*28.056–28.541*	7(*b*)
[2] *k* = 0.0035, *K* = −0.003 (recommended embedding parameters)	**50.003–50.006**	33.208*	8
[3] *k* = 0.001, *K* = −1.2	*53.058–56.157*	*37.509–37.556*	–
[4] *k* = 0.001, *K* = −2.5	*55.865–60.986*	*40.257–40.418*	–
[5] *k* = 0.001, *K* = −10.0	*66.156–77.272*	*51.127–51.351*	–
[6] *k* = 0.001, *K* = −50.0	*91.301–116.635*	**72.474–73.803**	9(*a*)
[7] *k* = 0.0015, *K* = −50.0	*83.188–100.236*	**66.615–67.524**	–
[8] *k* = 0.002, *K* = −50.0	*75.300–96.002*	**61.624–63.184**	–
[9] *k* = 0.0025, *K* = −50.0	*74.139–91.431*	*57.597*–**59.950**	9(*b*)
[10] *k* = 0.003, *K* = −50.0	Does not converge		–
[11] *k* = 0.004, *K* = −50.0	Does not converge		–
[12] *k* = 0.02, *K* = −50.0	Does not converge		9(*c*)

## Appendix A

### A1. Accessing the *Graph* *T*–*T* software

A web-based version of the *Graph* *T*–*T* software can be accessed at https://graphtt.github.io. The program code is open source and can be accessed at https://github.com/GraphTT/graphtt.github.io/ where a Zip file can be downloaded containing all component files required to set-up and run *Graph* *T*–*T* locally. This is done by clicking on the green ‘Code’ button and then by selecting ‘Download ZIP’. This Zip file (called ‘graphtt.github.io-main’) and instructions (called ‘GraphT–T Instructions rev.pdf’) about how to install and run *Graph* *T*–*T* locally are also available as supporting information. The open-source component code (see Section 3.2) can be accessed at github.com using the following links:

https://github.com/d3/d3-force

https://github.com/vasturiano/3d-force-graph

https://github.com/anvaka/ngraph.forcelayout.

If any problems are experienced when executing the open-source code or running *Graph* *T*–*T* locally, please contact Dr Ali Rostami (rostamiev@gmail.com) and Dr Maxwell C. Day (umday23@myumanitoba.*ca*).

### A2. Operating the *Graph* *T*–*T* software

Currently, graphs must be uploaded to *Graph* *T*–*T* in a G6 file format (*e.g.* graph.g6). Using the *GraphTea* program, graphs can be drawn and then saved as a G6 file, the G6 format string can then be entered into *Graph* *T*–*T*. *GraphTea* can be downloaded from http://graphtheorysoftware.com/ and a detailed description of how to use the software is provided by Rostami *et al.* (2014*b* ▸). The user can also test the program using the pre-loaded example graphs accessed using the ‘Generator’ dropdown menu. Examples ChainGraph1 and ChainGraph2 correspond to the chains observed in astrophyllite- and amphibole-supergroup minerals (*e.g.* Figs. 4 ▸ and 5 ▸), respectively. Examples ChainGraphs 4–7 are not observed in minerals and are embedded and evaluated by Day *et al.* (2024 ▸).

Once a graph has been loaded as a G6 file, one can start the embedding process by pressing the ‘Go’ button. Before embedding is started, the user may wish to adjust the embedding parameters accessed in the ‘Visualization’ dropdown menu. As discussed in Section 3.2.2, these parameters apply only to the second phase of embedding rather than the first phase of embedding in which each parameter is assigned a default value which cannot be adjusted by the user. It is advised that users use the recommended embedding parameters provided by Day *et al.* (2024 ▸) as a starting point in the refinement process of the embedding parameters (Section 4.3). At any point during the refinement process, the set embedding parameters can be saved using the ‘Local save’ button and then re-loaded using the ‘Load from local save’ button. If the browser is refreshed when using the web-based version of *Graph* *T*–*T*, the saved embedding parameters will be lost. During embedding, *Graph* *T*–*T* will report the minimum *T*⋯*T* separation distance and the minimum, maximum and average *T*–*T* distances for each consecutive iteration. Once the unit-distance graph has converged (if convergence is possible), R〈*T*–*T*〉 and R〈*T*⋯*T*〉_min_ can be recorded. Users may rotate or move the graph in the display interface using the left and right cursors and zoom in and out by scrolling. Additional options for reporting other properties of embedded unit-distance graphs and for exporting data (*e.g.* images and vertex coordinates) will be made available in the next version of the program (*Graph* *T*–*T* V1.1).
