# Supplementary material for: Graph T–T (V1.0Beta), a program for embedding and visualizing periodic graphs in 3D Euclidean space
Source: Acta Crystallogr A Found Adv. 2024 Apr 29;80(Pt 3):282–92. doi: 10.1107/S2053273324002523 (PMC11067947; doi:10.1107/S2053273324002523)
Supplement: Supplementary file 1 [file a-80-00282-sup1.pdf]

FOUNDATIONS
ADVANCES

Volume 80 (2024)

Supporting information for article:

GraphT–T (V1.0Beta), a program for embedding and visualizing periodic graphs in 3D Euclidean space

Maxwell Christopher Day, Ali Rostami and Frank Hawthorne

Instructions for local installation of GraphT-T (V1.0Beta)

All component files to set-up and run GraphT-T locally can be found in the Zip file "graphtt.github.io-main" which can be downloaded from:

https://github.com/GraphTT/graphtt.github.io/

On this page, a zip file can be downloaded by clicking the green button "Code". After unzipping the downloaded zip file and changing the user directory to the unzipped folder, the software can be run by simply clicking the file "index.html" and opening it in any available browser although, the firefox and google chrome browsers are recommended. By doing this the software is completely rewritten in JS and HTML to make it more accessible. A running instance is also available using the link https://graphtt.github.io/. Once GraphT-T is running, instructions on how to use the software can be found in the associated manuscript (Day *et al*. 2024, *Acta Cryst*).

If you have problems installing or running GraphT-T please contact Dr. Ali Rostami (rostamiev@gmail.com) and cc the corresponding author, Dr. Maxwell C. Day (umday23@myumanitoba.ca).
