# Supplementary material for: Graph T–T (V1.0Beta), a program for embedding and visualizing periodic graphs in 3D Euclidean space
Source: Acta Crystallogr A Found Adv. 2024 Apr 29;80(Pt 3):282–92. doi: 10.1107/S2053273324002523 (PMC11067947; doi:10.1107/S2053273324002523)
Supplement: Supplementary file 2 [file a-80-00282-sup2.zip › graphtt.github.io-main/index.html]

GraphT-T


### GraphT-T

Generators


G6 format


directed
undirected


Category: 
Select
  
Generator: 
Select

A brief description


n :

Existing matrices:  

No selection
  

Adjacency Matrix
Column-Intersection Graph
Row-Intersection Graph
Bipartite Graph
rho-Column-Intersection Graph
Special

G6 Format:

Select a file :

Force Directed
Circular
Grid
Preset
Botanical Tree

3D Force Directed
Load and Draw
Load Only
Fit Canvas
Clear Canvas
Canvas to Image
WebGL
Go


gen

Visualization Information:

---
