# Supplementary figures and images for: Graph T–T (V1.0Beta), a program for embedding and visualizing periodic graphs in 3D Euclidean space
Source: Acta Crystallogr A Found Adv. 2024 Apr 29;80(Pt 3):282–92. doi: 10.1107/S2053273324002523 (PMC11067947; doi:10.1107/S2053273324002523)

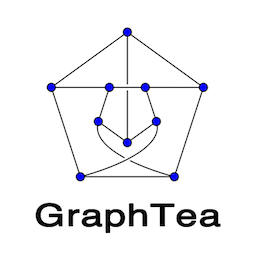

Supplement: Supplementary file 2 [file a-80-00282-sup2.zip › graphtt.github.io-main/icon-big.PNG]

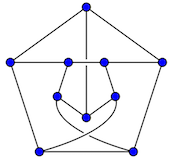

Supplement: Supplementary file 2 [file a-80-00282-sup2.zip › graphtt.github.io-main/icon.png]
